# A New Approach to Assessing HSV-1 Recombination during Intercellular Spread

**DOI:** 10.3390/v10050220

**Published:** 2018-04-25

**Authors:** Gabrielle A. Law, Alix E. Herr, James P. Cwick, Matthew P. Taylor

**Affiliations:** Department of Microbiology & Immunology, Montana State University, Bozeman, MT 59717, USA; gabriellealaw@gmail.com (G.A.L.); alixeherr@gmail.com (A.E.H.); jpcwick@gmail.com (J.P.C.)

**Keywords:** alphaherpesvirus, HSV-1, recombination, fluorescent protein, cell–cell spread, neuroinvasion, neuron culture, intravitreal injection

## Abstract

The neuroinvasive Herpes simplex virus type 1 (HSV-1) utilizes intergenomic recombination in order to diversify viral populations. Research efforts to assess HSV-1 recombination are often complicated by the use of attenuating mutations, which differentiate viral progeny but unduly influence the replication and spread. In this work, we generated viruses with markers that allowed for classification of viral progeny with limited attenuation of viral replication. We isolated viruses, harboring either a cyan (C) or yellow (Y) fluorescent protein (FP) expression cassette inserted in two different locations within the viral genome, in order to visually quantify the recombinant progeny based on plaque fluorescence. We found that the FP marked genomes had a limited negative affect on the viral replication and production of progeny virions. A co-infection of the two viruses resulted in recombinant progeny that was dependent on the multiplicity of infection and independent of the time post infection, at a rate that was similar to previous reports. The sequential passage of mixed viral populations revealed a limited change in the distribution of the parental and recombinant progeny. Interestingly, the neuroinvasive spread within neuronal cultures and an in vivo mouse model, revealed large, random shifts in the parental and recombinant distributions in viral populations. In conclusion, our approach highlights the utility of FP expressing viruses in order to provide new insights into mechanisms of HSV-1 recombination.

## 1. Introduction

Herpes Simplex virus type 1 (HSV-1) is a persistent and pervasive human pathogen with global relevance [1,2]. Infections of HSV-1 typically manifest in the mucosal or epithelial surfaces. Occasionally, infections can manifest on the corneal surfaces of the eye. Invariably, all HSV-1 infections will spread into peripheral neurons. Once infected, the peripheral neurons remain persistently infected with HSV-1 for the lifespan of the host, spreading the virus either back to sites of primary infection or into neurons of the central nervous system, where it can cause severe encephalitis [3,4].

Recombination is a primary driver of the genomic diversity and evolution of HSV-1. In order for recombination to occur, cells within the host tissues must become infected by multiple virions. The viral genomes are then able to exchange DNA during replication, in order to generate the recombinant genomes, which are packaged into progeny virions. There is substantial evidence to indicate that HSV-1 recombination occurs at significant rates during infection and replication [5,6,7]. The next generation sequencing of HSV-1 from patients demonstrates that the diversity of viral genomes correlates with geographical clades [8,9]. Interestingly, all of the analyzed genomes show evidence of extensive recombination, which is suggestive of high rates of co-infection in human populations [10]. Recombination can lead to a generation of strains that are more pathogenic and/or resistant to current anti-viral therapies. In fact, this inter-viral recombination is continuing to drive the alphaherpesvirus evolution. Chimeric genomes, resulting from recombination between HSV-1 and HSV type 2 (HSV-2), are circulating within patient populations [11]. The recombination between the wild-type and the attenuated vaccine strain of Varicella Zoster virus (VZV), a related alphaherpesvirus, has resulted in the generation of a new clade of VZV [12]. Knowing the frequency of recombination, as well as the factors which influence it, is an important part of understanding and treating the viral disease, and developing effective vaccines.

The mechanisms of inter-genomic recombination have been extensively studied for HSV-1 [13,14,15]. Recombination requires nuclear genome replication and occurs early during the replication of the HSV-1 double-stranded DNA genomes [16,17]. Despite all of this information, there have been issues regarding the definitive rates of HSV-1 recombination between in vitro and in vivo experimental models. It is clear that high rates of recombination occur in vivo, and that they can promote the development of novel, pathogenic isolates [18,19]. Many of these studies utilize viruses that are harboring mutations that facilitate the identification of parental and progeny genomes. While these mutations are useful for quantifying recombination, they often attenuate the virus to the point where its ability to replicate is considerably diminished, when compared to wild-type viruses. These attenuations inherently bias recombination experiments, so as to favor the replication of the non-mutant, non-marker bearing virus. To truly understand the rates and consequences of recombination, we will need to develop a system that does not utilize such strongly attenuated viruses to facilitate the easy, rapid detection of recombinant viral progeny following co-infection.

In the following study, we set out to engineer and characterize viruses with markers that did not significantly alter the kinetics of the infection, but would still allow for the accurate discrimination of parental and recombinant progeny. We did this by constructing fluorescent protein (FP), which expressed mutants that had limited attenuation compared with the wild-type HSV-1. Genetic cassettes bearing the FP markers were inserted at distant locations within the HSV-1 genome, at sites that were characterized as having a minimal effect on viral replication and fitness [20]. Following the characterization of viral replication and competition against wild-type HSV-1, we set out to analyze two major factors that contributed to intergenomic recombination: the rates of co-infection and intercellular viral transmission. To model the effect of the intercellular spread on recombination, we employed a combination of a sequential viral passage, a compartmentalized neuronal cell culture system, and an in vivo mouse model of the neuroinvasive viral spread.

## 2. Materials and Methods

### 2.1. Cells and Virus

Vero cells, a kind gift of the Enquist lab, were maintained in Dulbecco’s Modified Eagle’s Medium (DMEM), which contained 10% (*v*/*v*) fetal bovine serum and 1% penicillin/streptromycin. All of the steps of the viral isolation, analysis, and production were completed in Vero cells. All of the infections were performed with the HSV-1 strain 17, which was derived from a sequence verified isolate, provided by the Enquist lab. The viruses that were developed for the recombination expressing yellow fluorescent protein (YFP) with a nuclear localization sequence (NLS) (HSV-1 OK12, first described in [21] and referred to here in the manuscript as HSV-1 YFP_UL_) and cyan fluorescent protein (CFP)-NLS (HSV-1 MT002 referred to as HSV-1 CFP_US_ in the manuscript), which were both derived from the HSV-1 strain 17.

### 2.2. Construction of FP-Expressing Viruses

HSV-1 YFP_UL_ was a previously described virus, which contained a genetic cassette that was inserted between the viral genes U_L_37/U_L_38, which allowed for a cytomegalovirus (CMV) driven expression of a nuclear localization sequence that was tagged eYFP [21]. HSV-1 CFP_US_ was a novel recombinant strain, which was isolated for this work with a similar genetic cassette inserted between the viral genes U_S_1/U_S_2, however, this cassette encoded a nuclear localization sequence, which tagged Turquoise2. Both of the sites in the viral genome that were chosen for the insertion of this genetic cassette had been previously shown to tolerate genetic inserts without a reduction of viral titer [20].

The insertion of the Turquoise2 expression cassette required construction of a plasmid that contained a fluorophore expression cassette similar to HSV-1 OK12, which was flanked by homology arms that would promote the recombination into the U_S_1/U_S_2 region of the HSV-1 genome. Approximately 500 bp of the homologous sequence flanking the 5′ and 3′ of the Us 1/2 insertion site, was PCR amplified from purified HSV-1 strain 17 DNA. The Turquoise2 CMV driven expression cassette was PCR amplified from the plasmid vector, pMT06 [22]. All of the PCR amplification was done with a Q5 DNA polymerase mix (Qiagen, Hilden, Germany). The PCR products were visualized and isolated from a 1% agarose Tris-Acetate EDTA (TAE) gel followed by purification using gel extraction (Qiagen). All of the PCR products contained 15 bp of the homologous overlapping sequence in order to facilitate In-Fusion cloning (ClonTech, Mountain View, CA, USA) into a plasmid backbone, which contained ampicillin resistance. The generation of the final construct was achieved with a 3-way ligation reaction, followed by the transformation and isolation of resistant bacterial colonies. The positive recombinant clones were identified with a colony PCR. The resulting isolated and purified plasmid was verified by Sanger sequencing.

To isolate HSV-1 viruses with insertions in the U_S_1/U_S_2 locus, purified plasmids were linearized with an XmnI/XbaI double digest. The linearized plasmid was co-transfected with the purified HSV-1 strain 17 DNA into Vero cells. The resulting progeny plaques were screened for the Turquoise2 expression, followed by three subsequent rounds of plaque isolation in order to obtain high quality, stable, and pure isolates. All of the viral stocks were verified by fluorescence microscopy for maintenance of the FP expression. A minimum of 100 plaques were imaged to ensure a greater than 99% homogeneity of expression in the population of the viruses that were used for infections.

### 2.3. Microscopy

All of the fluorescent imaging was performed on a TiEclipse inverted fluorescence microscope (Nikon Instruments, Melville, NY, USA), which employed a motorized objective turret, motorized X–Y stage, and mechanical shutters for transmitted and fluorescent illumination. The fluorescence illumination was supplied by a SpectraX illuminator (Lumencor, Beaverton, OR, USA), which contained six high-intensity, band-pass restricted LED illuminators that were oriented for a single path of illumination. The excitation sources were paired with specific multi-pass dichroic mirrors and emission filters, including the CFP/YFP/RFP filter pairs (Chroma Technology Corp., Bellows Falls, VT, USA). To quantify the FP expression and frequency of phenotypes in the progeny viral populations, a multi-image tile was acquired from the viral plaque assays. For each image area, three channels were captured, which acquired a transmitted light, CFP, and YFP. The scoring of plaques was based on the FP expression as described below.

### 2.4. Quantification of Recombination

To assess the recombination frequency occurring during HSV-1 replication and spread, Vero cell monolayers were co-inoculated with equal amounts of HSV-1 CFP_US_ and YFP_UL_ at the indicated multiplicity of infection (MOI). Following a one-hour inoculation at 37 °C, the monolayer was washed with phosphate-buffered saline. The cells and supernatant that contained the progeny virions were harvested by scraping at the times the post-infection was indicated in each experiment. The infected samples were then subjected to one freeze–thaw cycle, ultra-sonicated, and titer evaluated by the plaque assay. The resulting plaques were imaged for the FP expression of individual plaques. The plaques that fluoresced only a single color were denoted as a parent genome and those that fluoresced two colors or those with no detectable fluorescence, were counted as a recombinant progeny.

The influence of the multi-cycle replication on HSV recombination was determined by passaging virion progeny from cells co-infected with the two FP-expressing viruses. The progeny from a Vero cell monolayer co-inoculated at an MOI of 10, of HSV-1 CFP_US_ and YFP_UL_, was harvest as described above. The progeny was plated at limiting dilutions onto cell monolayers in order to determine the viral titer and to quantify the marker expression in the plaques. This progeny, termed inoculum, was then used to infect the Vero cells at an MOI of 10. The resulting progeny of this first, and subsequently second and third, rounds of passage (a total of three sequential rounds of infection and amplification) was analyzed for viral titer and fluorescent examination in order to quantify the distribution of the plaque phenotypes. The statistical analysis of the resulting plaque phenotype distributions was performed in GraphPad Prism 7.0 software (GraphPad Software, La Jolla, CA, USA).

### 2.5. Compartmentalized Neuronal Cultures

Neuronal cultures were constructed with a trichamber design, as previously described [23,24]. Briefly, primary mouse superior cervical ganglions (SCG) were dissected from embryos, which were harvested at day 13.5 to 14.5 from pregnant C57Bl/6 mice. Plastic tissue culture dishes were coated with poly-ornithine (Sigma-Aldrich, St. Louis, MO, USA), followed by murine laminin (ThermoFisher Scientific, San Francisco, CA, USA), before culturing. Parallel groves were etched into the surface of culture dishes and covered by 1% methylcellulose in 1× DMEM. Autoclaved vacuum grease was applied to one side of CAMP320 Teflon three compartment rings (Tyler Research, Edmonton, Alberta, CA, USA). The Teflon rings were then placed on the tissue culture dishes so that the parallel groves were perpendicular to, and extended across all of the compartments. The dissociated SCG neurons were plated into one of the side compartments, which was labeled the cell body compartment. The SCG cultures were maintained in a neuronal media comprising of Neurobasal Media, which was augmented with 1% penicillin/streptomycin-glutamine, B27 supplement, and 50 ng/mL Neuronal Growth Factor 2.5S (ThermoFisher Scientific), until axons had penetrated and grown into the far axon compartment, which took approximately 17–25 days.

A monolayer of ~5 × 10^5^ Vero cells in 1% FBS supplemented neuronal media was plated in the far axon compartment one day prior to infecting the compartmentalized neuronal cultures. Before infecting the cell body compartment with the virus, the media in the middle compartment was replaced with 1% methylcellulose supplemented neuronal media. The compartmentalized SCG cultures were infected with 1 × 10^6^ pfu of each parent virus in conditioned neuronal media at the cell body compartment. Following the one-hour incubation, the inoculum was replaced with the original volume of media in the compartment. The cell body and axon compartments were both harvested after 48 h, followed by an evaluation of the viral titer and progeny marker expression, as described above.

### 2.6. Intravitreal Inoculation and Tissue Harvesting

The murine eye model was used to understand the influence of the HSV spread in neurons on diversity through recombination. We adapted the inoculation and tissue harvesting from the extensive work of J. Patrick Card [25,26]. Briefly, the murine eye model utilized the nervous system connections with defined retrograde and anterograde circuits in mice, in order to analyze the neuroinvasive spread. Primary inoculum was contained within the mouse’s eye, resulting in replication within the retinal cells. The virus spread to the brain occurs along synaptic connections with both the optic and oculomotor nerve fiber tracts.

All of the procedures with mice were submitted and approved through Montana State University’s Institutional Animal Care and Use Committee and conformed to the best practices, as defined by AALAC and the Office of Laboratory Animal Welfare guidelines. For all of the experiments, we used male mice between 8 and 12 weeks of age. The mice were anesthetized with isoflurane and ~1 × 10^6^ pfu (approximately 2 μL) of an equal inoculum of HSV-1 CFP_US_ and YFP_UL_ was injected intra-vitreally into the right eye. The infected mice were sacrificed after 72 h, using a pentobarbital solution (fatal plus) followed by cardiac perfusion with saline. Each skull was manually removed and the brain and infected eye were harvested and placed in Hank’s Balanced Saline Solution (HBSS) prior to processing. Each brain was dissected to isolate sub-regions, which reflected the different routes of spread. Briefly, each brain was placed in an adult mouse brain matrix (Zivic Instruments, Pittsburgh, PA, USA). First, two parallel slices were made at positions 6 and 12. Then, each brain was sliced in half to separate the left and right hemisphere. These slices allowed for the isolation of the two halves of the midbrain and the hindbrain. Based on the eye of injection, the midbrain sections were identified as being on the same side (ipsilateral) or the opposing side (contralateral). To obtain viral titers from the dissected tissue, we adapted a protocol from previously published methods [27]. Briefly, the dissected tissue sections were diluted in equivalent amounts of HBSS and snap-frozen in either liquid nitrogen or dry ice/ethanol, prior to storage at −80 °C. Following the rapid thawing, the tissues were homogenized by extensive trituration with a wide bore Pasteur pipet. The homogenized samples were ultra-sonicated and centrifuged at 1000 rpm in order to pellet large debris. The clarified supernatant was the source of the material that was used to assess the viral titer and quantification of progeny plaque phenotypes.

## 3. Results

### 3.1. Adapting Two-Color Fluorescent Protein Expression to Monitor Recombination

**Figure 1:**
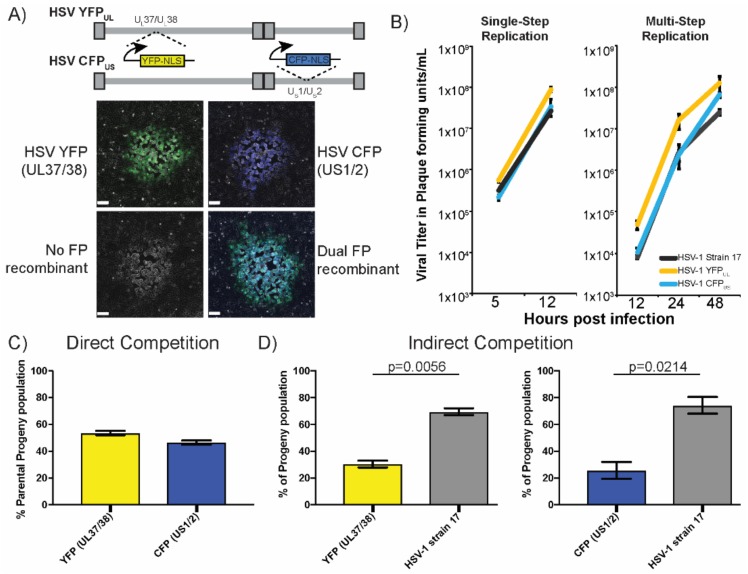
Characterization of the genetically tagged Herpes simplex virus (HSV) genomes. (**A**) Schematic representation of the fluorescent protein expression cassette location on the HSV-1 genome. Boxes represent large terminal and internal repeat sequences. Expression cassettes for yellow fluorescent protein (YFP)- and cyan fluorescent protein (CFP)-NLS proteins have been inserted in the UL36/37 and US1/2 loci, respectively. The genetic insertions result in different fluorescent expression phenotypes of the resulting viral plaques (images in panels below, scale bar = 100 μm). Parental infections result in YFP and CFP positive plaques. Upon co-infection, the genomes can recombine, producing progeny containing both of the genetic cassettes. Those plaques will either express both of the fluorescent proteins (Dual FPs) or the lack fluorescent protein expression (No-FP); (**B**) single step replication plots of parent viral isolates; HSV YFP_UL_, HSV CFP_US_, and HSV-1 strain 17. The proportion of the viral progenies were plotted following the (**C**) direct competition assay between parent viruses and the (**D**) indirect competition assay against HSV-1 strain 17. The indicated FP expressing HSV virus or HSV-1 strain 17 were co-infected at an MOI of 10 for each virus. The total infected cells were harvested at 8 hpi. The harvested virus was plated at a limiting dilution on the cell monolayers and plaques and was scored based on the fluorescent protein expression. A statistical *t*-test comparison of the direct and indirect competition assays revealed significant differences between the wild-type and FP expressing viruses.

To assess intergenomic viral recombination, we sought to develop a FP based marker transfer assay. We isolated two viruses that contained genetic cassettes expressing fluorescent proteins inserted at distant sites in the viral genome (Figure 1). These cassettes were inserted into the HSV-1 strain 17 genome, at sites that had been previously found to tolerate genetic insertions with limited impact on viral replication and spread [20]. The first virus contained an insert between the viral genes U_L_37/38 that drove the expression of a nuclear localized YFP (referred to as HSV-1 YFP_UL_). The second virus contained an insert between the viral genes U_S_1/2 that expressed a nuclear localized CFP (referred to as HSV-1 CFP_US_). The genetic distance between the insertion sites allowed for many potential recombination events to be visualized, based on changes in the FP expression. Co-infection with HSV-1 CFP_US_ and YFP_UL_ could result in four distinct viral progeny phenotypes, based on the FP expression in the viral plaques. The viral plaques that expressed either the YFP or CFP fluorescence were classified as progeny of parental genomes. Viral plaques that exhibited either a dual FP expression or no FP expression were classified as a progeny resulting from intergenomic recombination between the parental genomes (Figure 1A).

An important part of our analysis was the capability to rapidly and correctly identify plaque phenotypes. Plaques that expressed both of the FP’s could result from either recombination or from a mixture of multiple viruses amplifying in the same region of the cell monolayer. The distribution of the FP expression in the plaque could be used to discriminate the dual FP expressing viruses from the plaques that were initiated by multiple viruses (Supplemental Figure S1). We only observed mixed plaque phenotypes in approximately 1.5% of the population of the plaques, and all had revealed non-homogenous FP expression, which was often “sectored”. The frequency of mixed plaques was in line with prior observations that used mixtures of three HSV recombinants, each expressing a fluorophore in the same cassette. When assessing the distribution of plaques, we also observed that approximately 2% of plaques contained more than one fluorescent protein (an unpublished observation from [21]). Since these fluorescent protein cassettes were inserted in the same position, the only way to express the multiple fluorophores was from multiple viral genomes initiating the plaque. Importantly, the rate of the mixed plaque formation correlated with the concentration of the virus that was present in the diluted sample, plated onto cells for visualization. These parallel observations confirmed that the mixed plaques occurred infrequently and that the majority of the plaques were initiated by a single genome. Based on this interpretation, we continued to characterize the FP expressing viruses.

To assess HSV recombination, the parental viruses, HSV-1 CFP_US_ and YFP_UL_, needed to have similar capabilities of viral replication, or fitness, to each other. Ideally, these viruses would also maintain replication and spread capabilities that were comparable to the wild-type HSV-1. Initially, we compared the capacity of these viruses to replicate under conditions of single-step and multi-step replication (Figure 1B). The cells were inoculated with each virus at the indicated MOI’s, and were subsequently harvested at the indicated times, post-infection. The viral titer was assessed to measure the extent of the viral replication. We observed that the resulting viral growth kinetics were comparable for the three viruses and those viruses maintained the expression cassette during both the single and multi-step replication experiments. Viral fitness was further evaluated by looking at direct and indirect competition during mixed infections. The competition assays assess the fitness as a function of the viral ability to produce progeny when competing for resources. If two viruses have a similar fitness, they should have produced similar amounts of progeny. Direct competition was assessed by infecting cells with equal amounts of HSV-1 CFP_US_ and YFP_UL_. The resulting viral progeny were evenly distributed between the YFP and CFP expression, indicative of close to equal fitness between the two viruses (Figure 1C). Indirect competition was assessed by co-inoculating either HSV-1 CFP_US_ and YFP_UL_ with the parental HSV-1 strain 17. Under these conditions, there was an apparent reduction in both the CFP and YFP plaques, compared with the non-fluorescent plaques (Figure 1D). The resulting progeny were 70% non-fluorescent expressing, indicative of a moderately greater fitness of the wild-type virus, compared with the FP cassette containing viruses. Notably, both CFP_US_ and YFP_UL_ had the same apparent reduction in the amount of progeny when in competition with strain 17, which confirmed their equivalent relative fitness. Despite the reduced fitness of both viruses, these viruses demonstrated a significant improvement in fitness, with respect to previous studies on recombination. Importantly, the FP expressing viruses had an equivalent, if not slightly greater, capacity to replicate, compared with the wild-type viruses (Figure 1B). The direct and indirect comparison of fitness were not previously evaluated for the marked viruses that were used in prior recombination studies. The results indicated that a minimal impact was incurred on viral replication by insertion of either of the FP expression cassettes, which favored the continued experimentation with these new tools, in order to analyze the viral recombination.

### 3.2. Evaluation of Intergenomic Recombination

**Figure 2:**
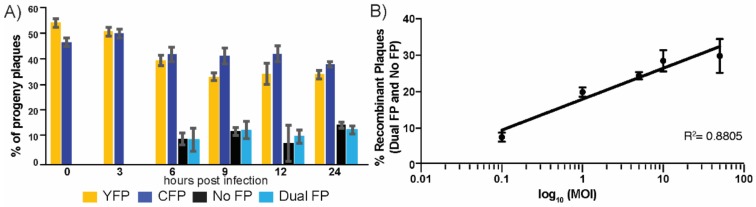
Effects of time and MOI on recombinant progeny output. (**A**) The effect of time during replication was assessed by counting the distribution of the fluorescent plaques during a time course of viral infection. Cells were infected at an MOI of 10 and subsequently harvested at 0, 3, 6, 9, 12, and 24 h post-infection. Infected cell lysates were plated at a limiting dilution and the resulting progenies were scored for the number of fluorescent plaques from each of the four genome types; (**B**) the effect of MOI on the total recombinant progeny production was assessed by differential inoculation. Vero cells were inoculated at an MOI of 0.1, 1, 5, 10, or 50 of the HSV YFP and HSV CFP viruses and the viral progeny was harvest at 8 hpi. As MOI increases, the frequency of the recombinant progeny increases with logarithmic progression. When the data are expressed as a plot of % recombinant progeny versus the log_10_ value of the MOI, the resulting linear slope is achieved with a logarithmic regression. Presented is a representative experiment involving replicates of three at each MOI that was evaluated, with standard deviation plotted between replicates.

Since there was little effect on the replication and progeny production of HSV-1 CFP_US_ and YFP_UL_, we proceeded to analyze the production of recombinant progeny during viral replication. To evaluate whether the amount of recombinant progeny that was produced was influenced by the time of the replication, we harvested cells over the course of a single replication cycle and assessed the progeny phenotypes by fluorescent microscopy. A balanced inoculum, which consisted of both HSV-1 CFP_US_ and YFP_UL_ at MOI 10 was used to infect cells. The cells were harvested at the indicated times, post co-infection, and the resulting plaque forming units were analyzed for FP expression. No recombinant progeny was detectable at 0 or 3 h post co-infection, which coincided with the eclipse phase of HSV-1 replication. The recombinant plaque phenotypes were detectable starting at 6 h post-infection, comprising 20% of the total analyzed plaque population (Figure 2A). This percentage of the recombinant plaques remained constant for the remainder of the collected time points. In addition to the recombinant progeny, all of the phenotypes remained at a relatively similar proportion of the population through the entire viral replication cycle. Our observations indicated that the production of recombinant progeny appeared to be constant throughout the course of viral replication. Importantly, we observed equivalent amounts of both recombinant phenotypes, which supported the argument that the FP-cassettes were not causing skewing of the recombinant progeny.

The ability to generate recombinant progeny was most likely influenced by the extent of cellular co-infection. The level of co-infection was positively correlated to the inoculating dose that was used during the experimental infections. We determined the influence of the inoculating dose on recombination by manipulating the MOI of the HSV-1 CFP_US_ and YFP_UL_ that were used to initially co-infect the cells. The cells were co-infected at the indicated MOI of infection, which was allowed to proceed for eight hours before the cells were harvested and the viral titer was quantified. The expression of the FP’s was assessed through the plaque assay and microscopy (Figure 2B). We observed that recombinant progeny increased as a function of the MOI of infection. At an MOI of 50, there appeared to be saturation, as the frequency of recombinant progeny only slightly increased. We also observed that at a low MOI of 0.1, there were significant amounts of recombinant progeny that were produced, making up approximately 7.6% of the total plaque population. Based upon the distribution of the recombinant progeny that was produced across the range of MOI’s, we chose to represent the data with a semi-log plot visualization, which revealed a near linear correlation between the recombinant progeny production, as a function of the inoculating dose. When taken together, the data supported the conclusion that the FP expression cassettes could be used to monitor recombination during HSV-1 co-infection. Further, our results were concordant with the previous reports on recombination rates and the timing of recombinant production during HSV-1 replication [13,14,15].

### 3.3. Stable Distribution of Recombinant Progeny during Sequential Passage

**Figure 3:**
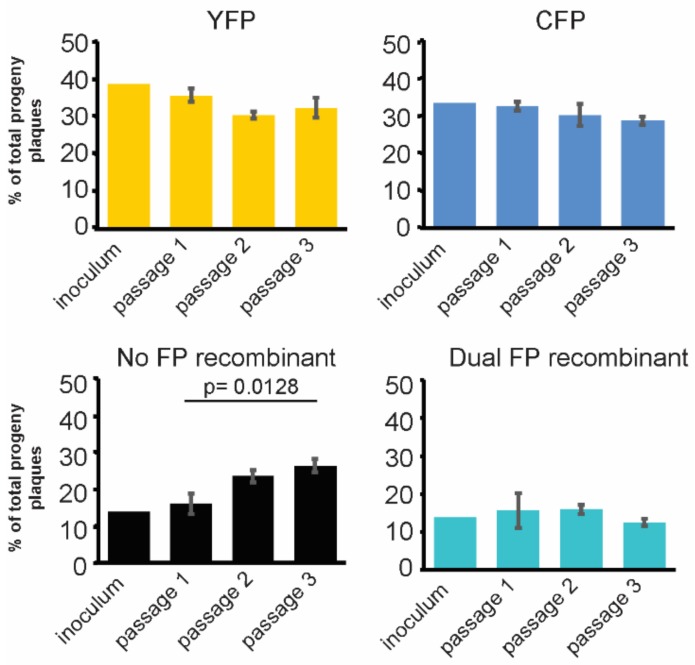
Recombinant progeny after multiple cycles of infection. A mixed population of HSV-1 viruses were generated following co-infection (inoculum) and progeny viruses were sequentially passaged three times. The inoculum and each subsequent passage were analyzed for content of the individual FP expression. Each plaque phenotype is plotted as a percent of the total plaque distribution. Data represents the average of the triplicate samples, with error bars as the standard deviation between replicates. Significant changes were only observed in the No-FP populations, as evaluated by one-way ANOVA.

Next, we wanted to understand whether the resulting recombinant progenies were influenced by intercellular spread. To address this question, we utilized sequential rounds of viral infection in immortalized epithelial cells, in order to understand changes in the viral populations. After the initial recombination events produced diverse populations, the viral population dynamics over several replication cycles were assessed by a serial passage of progeny. Following an initial co-infection with HSV-1 CFP_US_ and YFP_UL_, the progeny virus was sequentially passaged for three subsequent rounds of infection (Figure 3).

The viral populations that were sampled at each passage were assessed for the distribution of the FP expressing plaques. The initial population (inoculum) contained about 28% recombinant progeny, equally split between the dual FP-expressing and dark recombinants. At passage 1, the population contained approximately 32% recombinant progeny, with a slightly higher proportion of dark recombinants (16%) than the dual FP recombinants (15%). The total number of recombinants as a proportion of the population appeared to equilibrate at around 35%, by passage 2. Between the four plaque phenotypes, only the non-FP expressing population exhibited a slight, statistically significant, increase in the portion of the total viral population. The three other plaque phenotypes had equivalent slight decreases that were within the inter-sampling error between the replicates. Importantly, even after three serial passages, no single phenotype overtook a substantial proportion of the population. Parallel experiments using an MOI of 1 also produced similar results. These observations indicated that continued co-infection between the 4 possible genome configurations does not lead to dominance by any one viral genome, supporting our conclusion that the FP expression cassettes have limited effects on viral replication. Additionally, the apparent reductions of fitness that were observed during the indirect competition for the FP expressing viruses only resulted in a modest enrichment in the no-FP recombinant populations. This enrichment in no-FP recombinants was not detectable until the 2nd and 3rd passage of the population. These findings supported our conclusion that the FP cassettes were not skewing the resulting recombinant progeny populations and had a minimal effect on the replication and dissemination of these viruses.

### 3.4. Recombination during Transneuronal Spread

**Figure 4:**
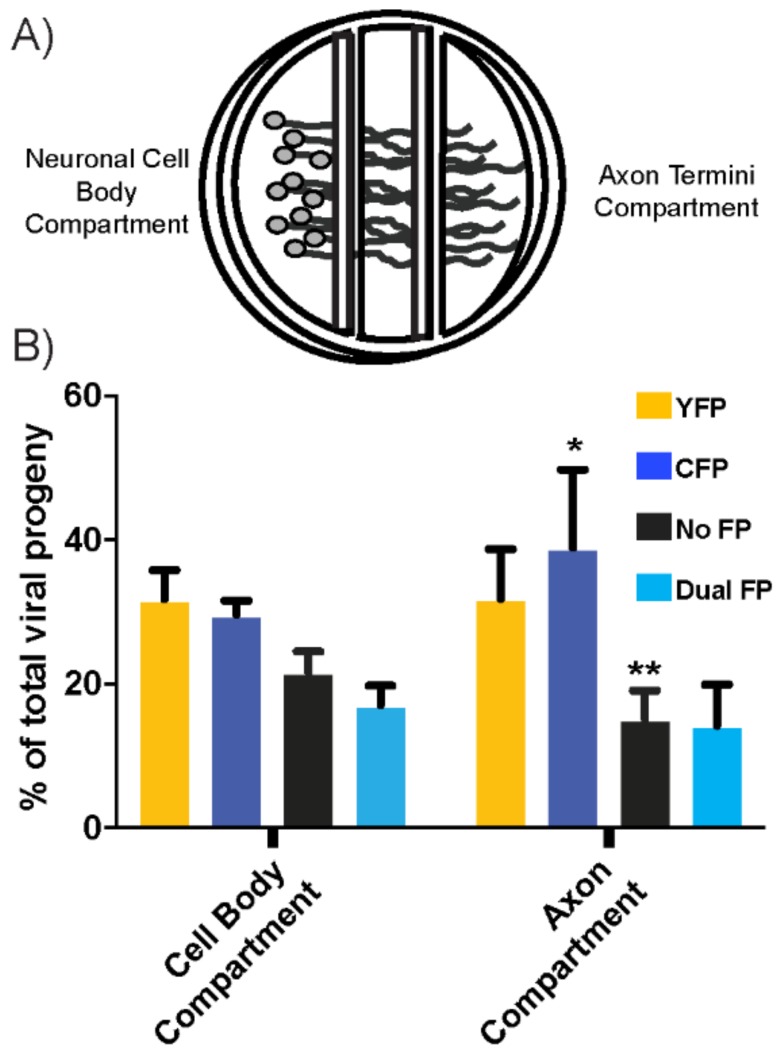
Effect of transneuronal spread on populations of recombinant viruses. (**A**) A schematic representation of the compartmentalized neuronal culture system. Briefly, a Teflon ring is attached to the culture surface. Dissociated superior cervical ganglions (SCG) neurons are plated in the left cell body compartment. Those cell bodies extend axon projections under the two internal walls, resulting in isolated axon termini in the Axon Compartment. Prior to infection, detector cells (not depicted in diagram) are plated in the axon compartment to amplify infectious progeny that transmit via axon-to-cell spread; (**B**) after 48 h of the cell body compartment inoculation, both compartments were harvested and plated at limiting dilution. The plaques developing from the viral progeny were analyzed for the distribution of FP expression in the cell body and axon compartments. * denotes *p* < 0.05, ** denotes *p* < 0.005 as identified through pairwise *t*-test comparison.

We were very interested to understand whether neuronal infection and spread had any impact on the recombinant progeny production. To address this concept, the recombination assay was implemented in a compartmentalized neuronal chamber, in order to determine the influence of neuronal replication and spread on the recombinant progeny production. The compartmentalized neuronal cultures were a model for neuronal infection and axonal spread of HSV-1 [23,24]. As depicted in the model diagram, the culture generated three distinct compartments, which separated the neuron cell bodies from the axon terminals, which allowed for the controlled inoculation of cell bodies and discrimination from the resulting axonal spread (Figure 4A). The Vero cells were plated on the exposed axons, to act as detector cells that received and amplified the infectious progeny. A balanced inoculum of HSV-1 CFP_US_ and YFP_UL_ was used to co-infect the neuronal cell bodies. The infection was allowed to progress for 48 h, at which point neuronal replication, axonal spread, and amplification in the detector cell layer had reached a maximum [21,24]. The progeny populations were plated at a limiting dilution and the plaque phenotypes were analyzed by fluorescent microscopy. Across a total of eight neuronal cultures, we consistently observed higher numbers of recombinant progeny in the neuronal cell body compartment compared with the axon compartment. On average, 38.6 ± 4.9 percent of the viral progeny in neuronal cell body compartments were recombinant. In the far axon compartments, where the infectious virions were transported and subsequently amplify in the detector cell layer, 29.3 ± 8.7 percent of viral progeny were recombinant (Figure 4B). Interestingly, there was a larger variance in phenotype distribution between replicates and the statistically significant shift in both CFP and No-FP populations in samples from the axon compartment. This variance was not related to differences in the total viral titer. One complication in interpreting these differences was the recombination that could have occurred during amplification of virus in the layer of detector Vero cells. However, this effect was likely minimal, based on the previous reports of limited viral co-infection following the axon-to-cell spread of the HSV-1 in this neuronal model [21]. We could conclude that the diverse viral populations that were generated in the cell body compartment transmitted the infection via the axonal spread to the epithelial cells, with only small changes in the composition and diversity of the viral population.

### 3.5. Recombinant Progeny Production during Transneuronal Spread In Vivo

**Figure 5:**
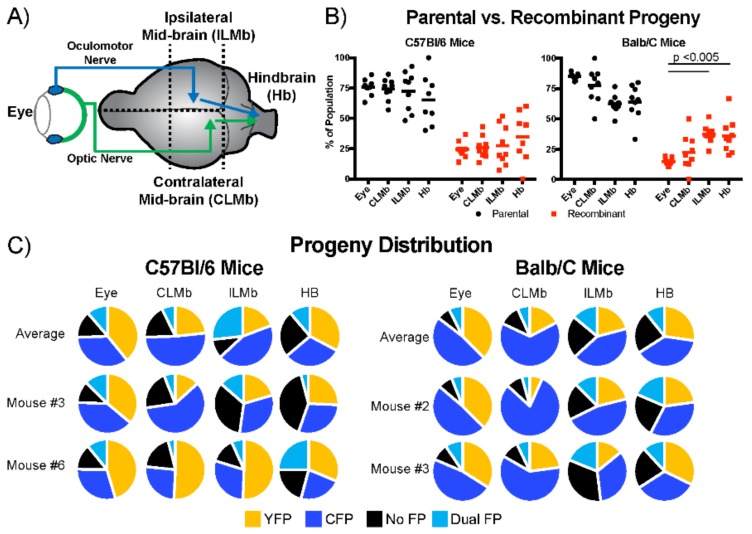
Effect of neuroinvasive spread on populations of viruses in vivo. (**A**) A schematic of the murine eye model for neuroinvasive spread; (**B**) frequency of parental progeny and recombinant progeny obtained from the eye, contralateral midbrain (CLMb), ipsilateral midbrain (ILMb), and hindbrain (Hb). Data presented are the summation of the two experiments for both C57Bl/6 and Balb/C mice. Each experiment contained between four and five mice; (**C**) distribution of FP distributions from the eye, CLMb, ILMb, and Hb are plotted. The top row shows the average distribution of plaques for the our sites in both C57Bl/6 and Balb/C mice. The lower rows are two example distributions from independent mice from each experimental group. Significant differences indicated were identified by two-way ANOVA analysis.

To observe the effect of trans-neuronal spread on recombination in vivo, we adapted our HSV-1 recombination assay to a mouse-eye model of neuroinvasive spread. When HSV-1 invades the central nervous system, infectious virions were transmitted between neurons at or near neuronal synapses. An effective method of modelling the central nervous system (CNS) invasion by HSV-1, was to use a murine model of the intravitreal viral inoculation. In this system, we were able to control the levels of primary co-infection and monitor the recombination rates as the virus spread from the eye into the CNS [26,28]. This system had the added benefit that all of the tissue was accessible, allowing the direct assessment of the viral populations at different stages of the spread in the mouse brain. The primary infection occurred within the cells of the ciliary body and in the retinal ganglion cell layers after the intravitreal injection into the right eye of the mouse. Following primary infection, progeny virions were transported within innervating nerve tracts. From the retinal ganglia cells the infection was transmitted via the optic nerve into the regions of the midbrain contralateral to the site of the injection. Similarly, from cells of the ciliary body the infection spread via the oculomotor nerve to the ipsilateral sites in the midbrain. Subsequently, the infection underwent one to two more synaptic transmissions before it reached neurons in the hindbrain (Figure 5A) [26,29].

The analysis of the recombinant viral populations following neuroinvasive spread was done on tissue that was extracted from the eye, the ipsilateral and contralateral midbrain, and the hind brain. To understand whether the host’s genetic background had influenced the potential outcomes, we compared infections in both Balb/c and C57Bl/6 inbred lines of mice, which exhibited differing susceptibilities to HSV-1 infection [30]. In both lines of mice, the primary replication in the eye exhibited distributions that roughly paralleled our results from the cultured cells, with between 20–25% of the viral progeny being the product of a recombination event. We observed more recombinant progeny as the virus spread deeper into the brain, which indicated a positive correlation between the number of the spread events and the relative proportion of the recombinant progeny in the population (Figure 5B). The amount of variability that was observed between the animals with respect to the diversity of viral populations also increased with trans-neuronal transmission events. There appeared to be more variation between the viral populations in the C57Bl/6 and Balb/c mice, as well as more differences in the production of the recombinant progeny. In the Balb/c mice, the eye and the contralateral midbrain (CMb) populations had a similar frequency of recombinant progeny. However, the frequency of recombinant progeny was significantly different in the ipsilateral midbrain and hindbrain sites (ILMb and Hb, respectively). There was an increased variability in the parental and recombinant genotypes between the individual mice and between the sites of the infection in the same mouse (Figure 5C). On average, the viral populations in the C57Bl/6 mice favored the expansion of the no-FP viral populations, especially in the hindbrain. In contrast, the Balb/C mice supported higher levels of the CFP_US_ virus. Curiously, the variation in the distribution of the viral populations at different sites in the same animal were very large. From these distributions, we concluded that the changes to the viral populations were greater than what was expected from the sequential passage or the single transneuronal spread experiments. Importantly, for this method, there was no apparent selective advantage that was driving the outgrowth of the viruses that lacked the FP expression cassettes. The sum of our observations supported our rationale for using limited attenuated viruses in order to track recombination in the various models of the intercellular spread.

## 4. Discussion

We set out to analyze the recombination during HSV-1 infection using viruses that contained the FP expression cassettes, which would have little detrimental effect on viral fitness. Using two viruses that maintained high levels of relative fitness, we found that the extensive recombination between genomes produced upwards of 30% of the viral progeny expressing recombinant phenotypes. The mixed populations of the viruses that were generated by recombination, revealed a limited change during the sequential passage and during one round of transneuronal spread. Interestingly, distributions of viral populations changed dramatically during neuroinvasive spread in an animal model of infection. We concluded that the FP expressing viruses were a new way to monitor recombination mediated change in mixed viral populations. Further studies could employ pairs of viruses that harbor the FP cassette at differing genomic sites or that contained it on different genomic backgrounds. Additionally, an analysis of the selection pressure effects could take advantage of closely paired FP genetic markers.

One of the biggest goals of our research was the development of marked viral genomes with limited attenuation on the viral replication or spread. The added benefit of using FP expression was the simple and rapid discrimination between parental and recombinant viral progeny. While the measurement of the viral replication did not reveal any differences between the FP expressing viruses, the indirect competition of the two viruses against the parental HSV-1 strain 17 did reveal reductions in competitive fitness. It is worth noting that the differences in the replication were amplified because of the nature of the analysis; each gain of one virus came at the detriment to the other. In fact, the sequential passaging of the mixed viral populations suggested there was only a small fitness advantage that was afforded to the genomes that lacked the FP expression cassettes. It was also not surprising, given that the added genome content of about 3 kb of DNA per genetic cassette, that each of the FP expressing viruses would influence the replication and virion assembly [20,31]. Furthermore, the evaluation of the mixed viral populations in the neuronal models of the infection did not reflect any strong pressure against the maintenance of the genetic cassettes. Together these data indicated that the insertion of the FP expression cassette had limited influence on viral replication and was not unfavorably skewing the results of our experiments in order to analyze recombination.

The overall extent of recombination that was observed and the independence of the recombinant progeny from the time during the viral replication were completely in line with prior observations [32,33,34]. Additionally, the dependence of the recombinant output on the MOI was also in line with prior observations and expectations. Interestingly, the frequency of recombinant progeny as a function of the MOI resulted in a surprisingly close linear response when it was modeled as a logarithmic function. At the lower end of the MOI range we observed a surprisingly high rate of recombinant progeny production. The extent of the recombinant production at an MOI of 0.1 was in line with the expected extent of the co-infection, based on the Poisson-based distributions of co-infection. In contrast was the apparent saturation of recombinant progeny production at the highest amounts of viral inoculum. The saturation could be best explained by the apparent limitation on the number of viral genomes that could replicate in a cell [35,36]. As the MOI exceeded the carrying capacity of the cell for viral genomes, so too would the capability to produce the recombinant progeny be limited. Together, the variables of the co-infection and limitations on the viral genome replication were both influencing the rate of the recombination in our experiments.

An unfortunate limitation of our approach was the inability to calculate an absolute rate of recombination, during HSV-1 replication. Calculating the rate of recombination would have required some assumptions that were not supported by prior research. Firstly, we would need to know the absolute distance between the two genetic markers. However, the ability of the HSV-1 genomes to change the relative orientation between the U_L_ and U_S_ regions leads to four different genome isomers [37]. The resulting genetic distance between our two genetic cassettes then ranged from as little as 50 kb to upwards of 110 kb. The second assumption was that recombination occurred at an even rate across the viral genome. However, prior research suggested that so called “hot-spots” for recombination correlate with small and large repetitive DNA elements scattered throughout the HSV-1 genome [19]. The largest limitation was in the assessment of the recombinant progeny. The assay that we developed only detected recombination upon the exchange of the expression cassette; we were unable to detect the recombination reactions outside of the two cassettes or as the result of a double cross-over event between the cassettes. It was even more complicated when analyzing the sequential passage and neuroinvasive spread data. After the initial co-infection produced the recombinant progeny, that progeny underwent co-infection and recombination with the myriad combinations of the viral genomes. The high rate of the recombinant progeny that was produced from the first cycle of the co-infection would then have influenced the reversion of the genomes back to the parental phenotypes at an equivalent rate to the production of new recombinant phenotypes. Despite an inability to calculate an absolute rate of recombination, there were many advantages to the approach we took that could continue to provide insights into HSV-1 recombination.

The strategy of the fluorescent marker transfer to analyze HSV-1 recombination already highlighted one outstanding issue in the field. By combining our fluorescent markers with an in vivo model of the neuroinvasive spread, we were able to observe the same larger apparent rates of recombination that others had reported for in vivo infections [5,19,38,39]. The difference was that our FP expressing viruses were comparably fit, with an equal capacity to infect and replicate. A comparison of the phenotype distributions during the neuronal infection and spread did not reveal major changes that would unduly skew the interpretation of the recombinant progeny. The relative similarity was likely why we saw that the initial production of the recombinant progeny at the sites of injection in mice mirrored our results from cultured cells. The resulting distributions of FP phenotypes diverged from our cultured experiments as the mixed viral populations spread into the brain. After undergoing multiple cycles of neuronal replication and transneuronal spread there was a great divergence between the individual samples, which became most amplified in the hindbrain. The apparent enrichment of No-FP viruses in the C57Bl/6 mice potentially mirrored the enrichment that we observed during the sequential passage. In contrast, the enrichment of the CFP_US_ virus in the Balb/C mice may have reflected some gain in the neuronal infection, replication, or spread, which was moderately indicated in the compartmentalized neuronal cultures. Intriguingly, multiple animals supported the recombinant plaque populations that exceeded 50% of the total virion population. This enrichment in recombinant progeny was most likely due to the stochastic selection processes that were enforced on the population of viruses that were transmitted between neurons. Limitations on the extent of the co-infection and on transneuronal spread of viruses were observed in a number of in vitro and in vivo systems [21,40]. With this assay, it was now possible to identify the effect and potential mechanisms of such stochastic selection on viral evolution.

There is great potential for the strategy of genetically tagged genomes in order to produce new findings in the HSV-1 recombination. Despite the limitation that one pair of markers cannot elucidate the true rate of recombination, a strategy employing multiple different pairs of FP cassette containing genomes would be able to overcome many of the limitations and assumptions. Similarly, we can pair the fluorescent cassettes with the attenuating mutations to evaluate how recombination drives viral evolution in different infection models. The pair of viruses we have developed can already be exploited as an easy read-out for recombinant progeny, in order to evaluate the effect of gene disruption or chemical inhibition on viral and cellular pathways, which are hypothesized for their importance in HSV-1 recombination. In summary, we have established a strong foundation for the continued development of viral genomes harboring FP expression cassettes, in order to analyze HSV-1 recombination.

## Supplementary Materials

Supplementary materials can be found at http://www.mdpi.com/1999-4915/10/5/220/s1.
