# Supplementary material for: A New Approach to Assessing HSV-1 Recombination during Intercellular Spread
Source: Viruses. 2018 Apr 25;10(5):220. doi: 10.3390/v10050220 (PMC5977213; doi:10.3390/v10050220)
Supplement: Supplementary file 1 [file viruses-10-00220-s001.pdf]

Supplemental Material

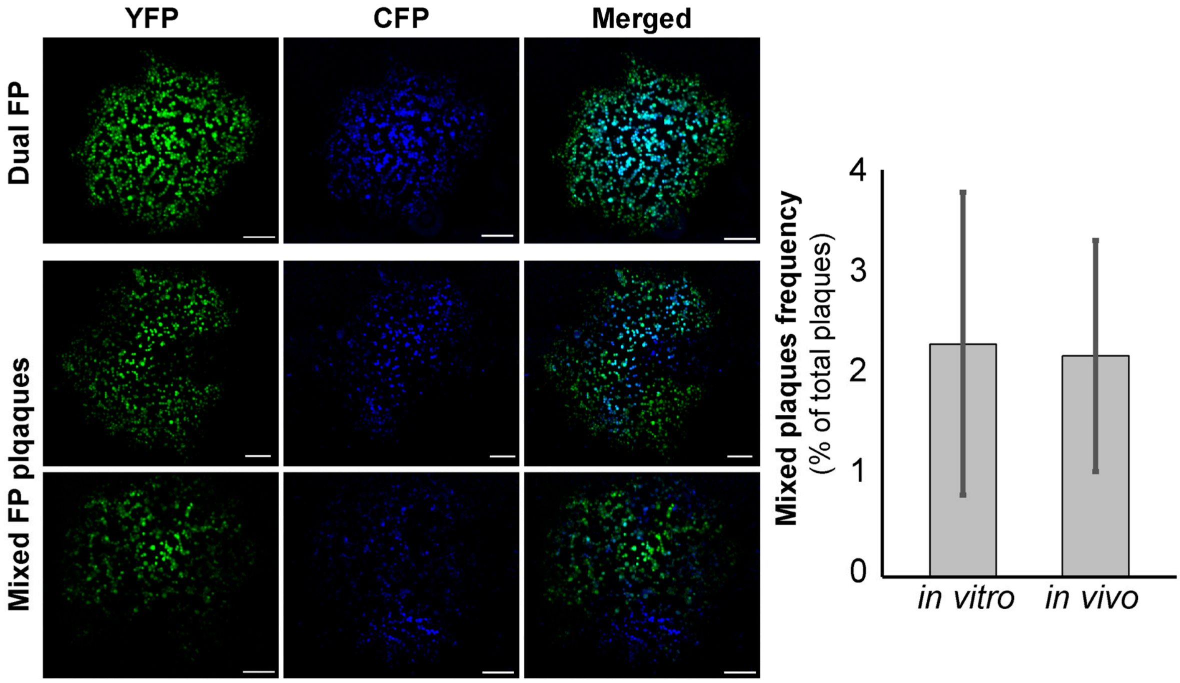

Figure 1. Discrimination and frequency of mixed plaque phenotypes. Plaques from progeny that express dual FP markers are visually distinguishable from plaques initiated by multiple virions expressing different FP's. (A) Representative images of dual FP plaques. The fluorescence of each FP is expressed equally within the plaque. Detection of CFP associated fluorescence lags behind detection of YFP expression based on relative expression of the two FP expression cassettes. (B) Representation of mixed FP plaques. There is unequal fluorescent expression of FP across the plaque resulting in sectored FP expression, indicated by white dashed lines. Scale bars for all images represent 200 m. (C) Frequency of mixed plaques observed (as percent of total plaques) during titering of samples produced during in vitro or in vivo samples. Average value is plotted from 3 samples of each type where between 150-500 plaques total being counted in each well. The standard deviation between samples is plotted.
